# High Mg# of the continental crust explained by calc-alkaline differentiation

**DOI:** 10.1093/nsr/nwac258

**Published:** 2022-12-07

**Authors:** Ming Tang, Xuanyu Liu, Kang Chen

**Affiliations:** Key Laboratory of Orogenic Belt and Crustal Evolution, Ministry of Education; School of Earth and Space Sciences, Peking University, Beijing 100871, China; Key Laboratory of Orogenic Belt and Crustal Evolution, Ministry of Education; School of Earth and Space Sciences, Peking University, Beijing 100871, China; State Key Laboratory of Geological Processes and Mineral Resources, China University of Geosciences, Wuhan 430074, China

**Keywords:** high-Mg# andesite, calc-alkaline differentiation, Fe depletion, slab melting, continental crust

## Abstract

We used compiled geochemical data to investigate the mechanisms that control Mg# (molar ratio of Mg/(Mg + Fe_T_)) in andesitic arc lavas. We find that andesites from mature continental arcs with crustal thickness of >45 km have systematically higher Mg# than those from oceanic arcs with crustal thickness of <30 km. The elevated Mg# in continental arc lavas results from strong Fe depletion during high-pressure differentiation favored in thick crusts. This proposal is reinforced by our compiled melting/crystallization experiment data. We show that the Mg# characteristics of continental arc lavas match that of the continental crust. These findings suggest that the formation of many high-Mg# andesites and the continental crust may not require slab-melt/peridotite interactions. Instead, the high Mg# of the continental crust can be explained by intracrustal calc-alkaline differentiation processes in magmatic orogens.

## Teaser

A new hypothesis based on global volcanic rock data links the enigmatic high Mg/Fe ratio of the continental crust to magma differentiation beneath high mountains.

## INTRODUCTION

The classical model for the continental crust formation emphasizes oceanic arc magmatism, which produces voluminous felsic magmas necessary to account for the andesitic average composition of the continental crust [1,2]. But on the other hand, the continental crust possesses an Mg# of >0.5, which is significantly higher than those of most andesites found in oceanic arcs. This discrepancy has led to a search of processes other than intracrustal arc differentiation to explain the composition of the continental crust [e.g. 3–5].

Over recent decades, much effort has been devoted to identifying slab-derived andesites as a potential high-Mg# candidate. Subducting slab may undergo dehydration and/or partial melting, depending on the P–T conditions of the slab surface [6,7]. We refer to slab-derived andesites specifically as those originating from slab melting; the resultant silicic melts would then interact with mantle peridotites and acquire high Mg# as they ascend through the overlying mantle [5,8–12]. These andesites may have sufficiently high Mg# (>0.5) and solve the Mg# problem of the continental crust if they constitute a significant component of the crust [e.g. 3,4,13–15]. If, indeed, this unusual type of andesite represents a major building block of the continental crust, the formation of the continental crust would require very specific subduction settings that favor slab melting, which are rare on modern Earth [6].

Here we use compiled data to investigate how crustal thickness influences the Mg# of arc magmas as they differentiate in the crust. We emphasize the role of crustal thickness because crustal thickness dictates the pressure of intracrustal differentiation, which in turn exerts important controls on the composition of evolved arc magmas [16–21]. We then use our findings to revisit the question of whether intracrustal differentiation alone can generate felsic magmas with sufficiently high Mg# to match that of the continental crust.

## DATA SOURCES

For arc lava samples, we took the compilation data set assembled by Ref. [22]. This data set contains 36 947 volcanic rock samples of Pleistocene to Holocene ages from global arcs. We divided the data into two groups. Group 1 includes samples from thin-crust arcs (10–30 km, crustal thickness converted from elevation), which are mostly oceanic arcs; Group 2 has samples from thick-crust arcs (45–80 km), which are manifested as mature continental arcs. Groups 1 and 2 represent two endmembers of global arcs based on arc crustal thickness. This division of the data set is arbitrary. The purpose of this division is not to capture the entire compositional spectrum of all arcs, but to articulate the compositional differences between thin-crust and thick-crust arc endmembers.

We also compiled partial melting/crystallization experiment data. We did not separate partial melting and crystallization processes because they are chemically equivalent in general. We divided the experiment results into a low-pressure group (0–1.0 GPa) and a high-pressure group (1.0–2.0 GPa). To be consistent, we excluded experiments using alkali basalts as starting materials as they are unlikely to be the parental magmas or source rocks of most arc felsic magmas. Most of the experiments were conducted under oxygen fugacities > fayalite-magnetite-quartz buffer (FMQ) (Supplementary materials).

## RESULTS AND DISCUSSION

### Crustal thickness control on arc magma FeO_T_ content and Mg#

Mg# is a function of both Mg and Fe contents, so an increase in magma Mg# can result from either Mg addition or Fe depletion. It is well known that arc magmas are on average depleted in Fe compared with those in mid-ocean ridges, but it is very underappreciated that the extent of Fe depletion varies significantly even within arcs. In particular, continental arc magmas are systematically more calc-alkaline and thus Fe-depleted than oceanic arc magmas [18,22–25].

**Figure 1.**
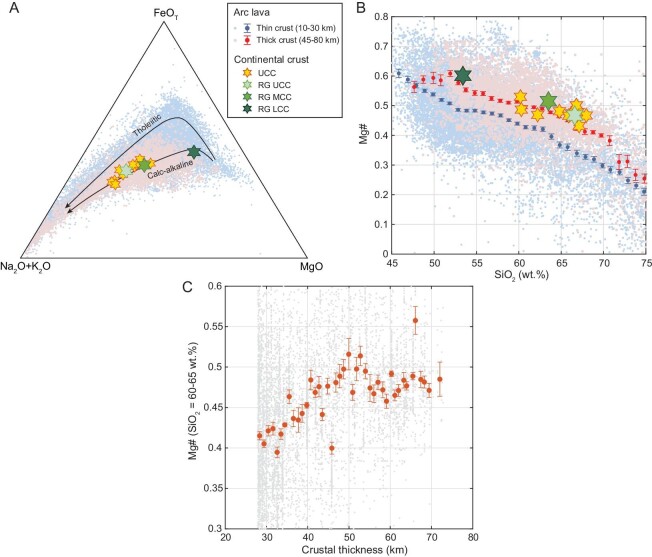
(A) AFM (K_2_O+Na_2_O−FeO_T_−MgO) diagram showing the compositional evolution of lavas erupted in thin- and thick-crust arcs. (B) Mg# evolution of arc lavas. To show the systematic difference between thin- and thick-crust arc lavas, we also plot the binned averages with two standard errors. Bin width 1 wt% SiO_2_. For comparison, we plot the compositions of the upper, middle and lower continental crust estimated by Ref. [26] and various other estimates of the upper continental crust composition [27–37]. (C) Correlation of Mg# of arc lavas with SiO_2_ = 60–65 wt% with crustal thickness. Bin width 1 km.

One major difference between continental arcs and oceanic arcs is crustal thickness, with continental arcs having thicker crusts. The influence of crustal thickness reflects the effect of pressure on magmatic differentiation. In thin-crust arcs (10–30 km) or oceanic arcs, FeO_T_ content in magmas shows pronounced enrichment before declining in late stage (tholeiitic trend); in thick-crust arcs (45–80 km), or continental arcs, FeO_T_ content shows continuous depletion through nearly the entire differentiation range (calc-alkaline trend) (Fig. 1A).

What causes Fe depletion in arc magmas remains a contentious question. Many early studies suggested that Fe depletion in calc-alkaline differentiation results from early magnetite fractionation due to the oxidized and hydrous compositions of arc magmas [38–40]. However, this magnetite hypothesis cannot explain why Fe-depleting differentiation preferentially occurs in continental arcs. This is because high-pressure intracrustal differentiation in thick crusts strongly suppresses magnetite saturation as shown by both thermodynamic simulations [23] and experimental studies [41,42]. One additional difficulty with the magnetite hypothesis is that magnetite fractionation strongly depletes Fe^3+^ in the derivative melt and limits further magnetite fractionation. Thereby, a high initial oxygen fugacity typical of arc magmas is not enough; continuous magnetite fractionation requires a sustained influx of oxidants throughout magmatic differentiation, which is not supported by any observations so far.

An alternative hypothesis for Fe depletion argues for the role of Fe-rich silicate minerals, particularly garnet [18,43–45]. One major advantage of the garnet hypothesis is that garnet fractionation is favored at high pressures and high water contents. Although arc magma differentiation may involve complex interplay between crystal fractionation, assimilation and magma mixing, garnet (±amphibole) fractionation is critical and provides a straightforward explanation for the correlation between the extent of Fe depletion and crustal thickness. Recent work on Fe isotopes in andesites further supports the fractionation of garnet (±amphibole) as the primary driver of Fe depletion in arc magmas [46].

If one realizes the pressure effect on Fe, it becomes obvious that magmas formed in arcs of different crustal thicknesses should be treated separately when studying their Mg#. Our arbitrary division of the global arc lava data set shows that samples from thick-crust arcs have systematically higher Mg# than those from thin-crust arcs (Fig. 1B). The correlation of andesite Mg# with crustal thickness is further shown in Fig. 1C, in which andesite Mg# increases as crustal thickness increases from 30 to 50 km. Note that the average trend of thick-crust arc lavas overlaps with the compositions of the upper, middle and lower continental crust (Fig. 1B). This observation would suggest that high-pressure intracrustal differentiation, facilitated by synmagmatic crustal thickening, can generate felsic magmas with sufficiently high Mg# to match that of the continental crust.

**Figure 2.**
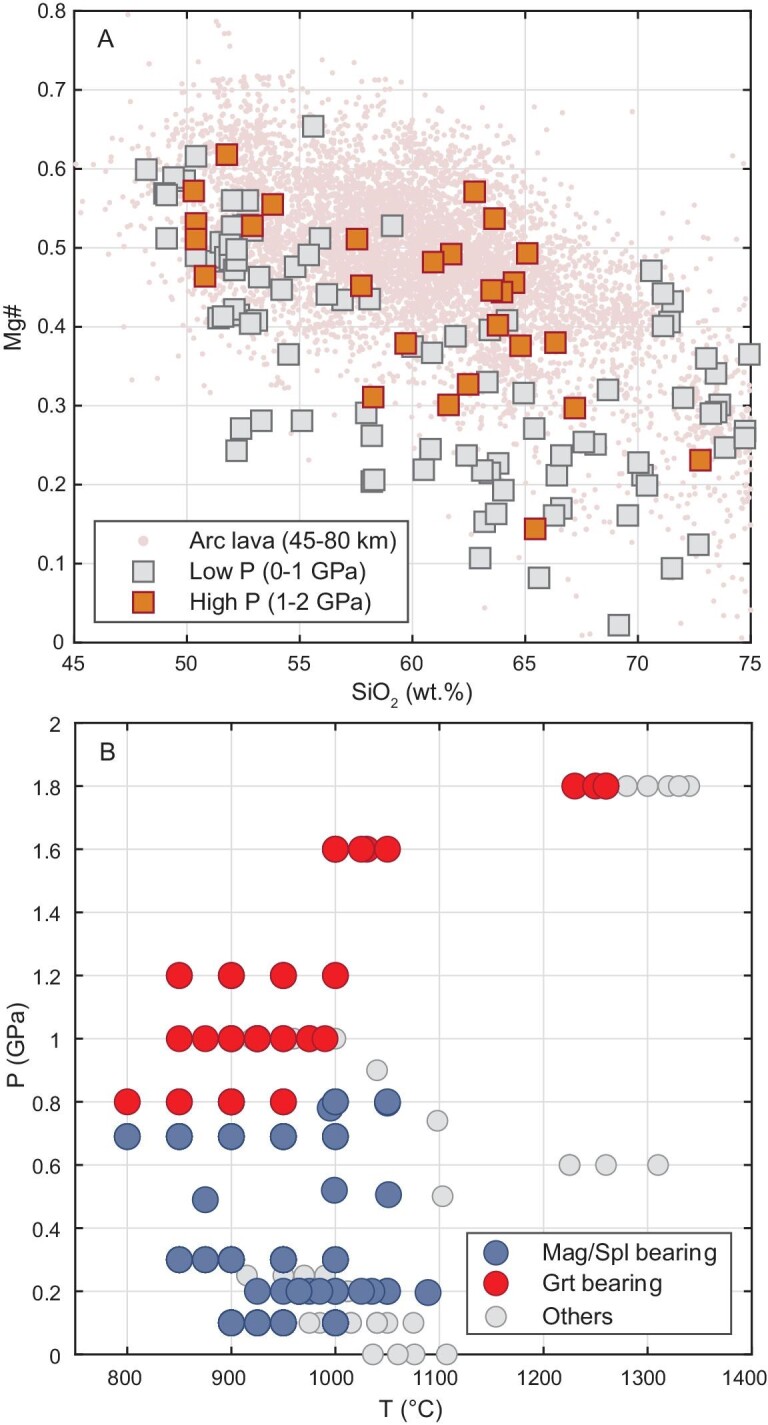
(A) Mg#–SiO_2_ relationship for experimental melts generated under high and low pressures. We only plot experiments using basaltic starting materials in this panel. Natural samples from thick-crust arcs (45–80 km) are also plotted here for comparison. (B) Occurrences of magnetite/spinel (Mag/Spl) and garnet (Grt) in experiment products formed under various pressures and temperatures.

The pressure effect on magma Mg# is also supported by crystallization/partial melting experiments. In Fig. 2A, we only plotted the results of experiments conducted using basalts (according to the data sources) as starting materials. Experiments using differentiated starting materials may result in relatively low Mg# of the derivative melts. One such example is the study by [45]. The starting materials used by [45] have andesitic bulk compositions with Mg# of 0.40. This led to low Mg# of 0.15–0.42 of their derivative melts. The experiment data are somewhat scattered, possibly due to the compositional differences in starting materials, redox conditions and experimental/analytical artifacts. Nevertheless, partial melts generated at high pressures (1–2 GPa) have systematically higher Mg# than those generated at low pressures (0–1 GPa) (Fig. 2A). Note that garnet is a common residual/crystallizing phase in high-pressure experiments, whereas magnetite is only present in experiments run under 1 GPa (Fig. 2B). These experiment data clearly show that high-pressure differentiation involving the fractionation of garnet, not magnetite, is efficient in increasing Mg# of the derivative melts. More importantly, the andesitic melts produced by high-pressure experiments broadly overlap with andesites from thick-crust arcs in this Mg#–SiO_2_ plot.

### Limited contributions from slab melting in continental arc lavas

What is the role of slab melting in generating the calc-alkaline lavas with high Mg# in continental arcs? Under appropriate conditions, subducted oceanic slabs may undergo partial melting in the mantle and produce silicic melts, which upon interaction with mantle peridotites may generate high-Mg# andesitic magmas [5,8–11,47]. Although high-pressure intracrustal differentiation is able to generate high-Mg# felsic magmas in continental arcs, is it possible that continental arc magmas also owe their high Mg# to significant inputs of slab melts equilibrated with peridotites? Insights to this question may come from the Andean arc.

**Figure 3.**
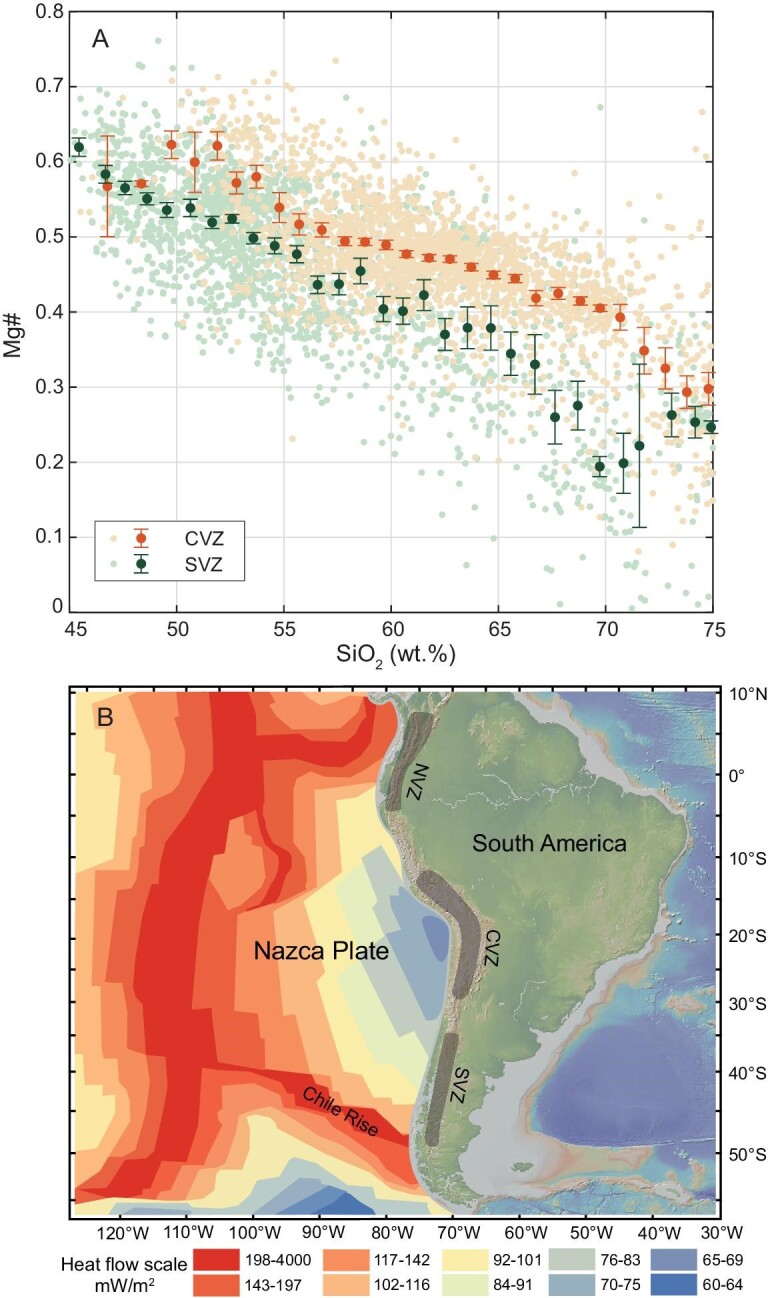
(A) Mg#–SiO_2_ relationship for Central Volcanic Zone (CVZ) and Southern Volcanic Zone (SVZ) lavas in the Andean arc. Similar to Fig. 1B, the data are binned by SiO_2_ content (bin width = 1 wt%) and the mean values and two standard errors are shown. (B) Map showing the locations of Northern Volcanic Zone (NVZ), CVZ and SVZ. Left half of the map is overlain by a heat flow map from Ref. [52].

The Central Andes, or the Central Volcanic Zone (CVZ), experienced substantial crustal thickening over the last ∼25 Ma, reaching the modern thickness of 60–80 km [48]. The Southern Andes, or the Southern Volcanic Zone (SVZ), developed on a thinner continental crust of ∼40 km [49]. The CVZ trend shows systematically higher Mg# than the SVZ trend (Fig. 3A). Slab melting requires hot geothermal gradients. In the Phanerozoic, such a condition is only met when the subducting slab is very young and thus warm [50,51]. If the elevated Mg# of the CVZ trend were due to the addition of peridotite-contaminated slab melts, one would expect the subducting plate beneath CVZ to be hotter. However, the opposite is true. The Nazca Plate currently subducting beneath CVZ exhibits a heat flow of 65–75 mW/m^2^, which is significantly lower than that of the plate subducting beneath the SVZ (Fig. 3B). The hotter plate subducting beneath the SVZ is associated with the Chile Rise—a divergent plate boundary extending from the triple junction of the Nazca, Pacific and Antarctic plates to the Southern Andes. Because we only consider lavas of Pleistocene to Holocene ages here, we do not expect the slabs subducted in the last few million years to be significantly different from those approaching the trenches today. These findings suggest that most high-Mg# andesites formed in continental arc settings are not associated with slab melting.

**Figure 4.**
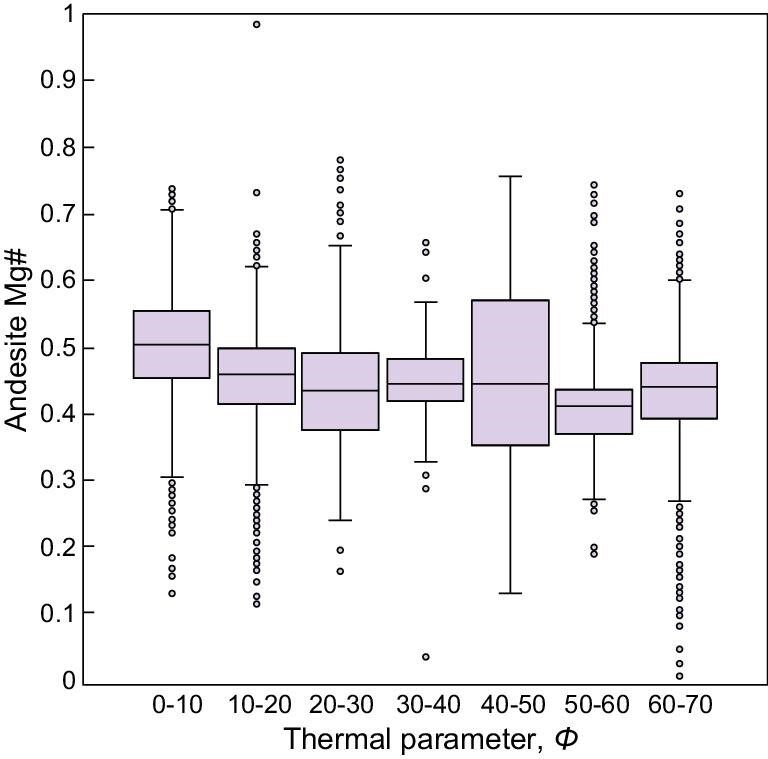
Box and whisker plot showing the relationship between andesite Mg# and subduction thermal parameter. The ends of the boxes show the upper and lower quartiles, and the ends of the whiskers show the 2.5 and 97.5 percentiles. Outliers are plotted as individual points.

A survey of global andesite Mg# and slab temperature provides additional evidence to evaluate the role of slab melting in arc volcanism. The thermal parameter *Φ* = t*v*sin*θ*, where *t* is age, *v* is slab subducting speed and *θ* is slab angle, has been frequently used as a proxy for the internal temperature of subducting slabs [53]. We find no clear correlation between andesite Mg# and subduction thermal parameter (Fig. 4). Because hot slabs are more likely to melt as they subduct, this lack of correlation suggests negligible contributions from slab melting to arc volcanism, at least on a global scale.

**Figure 5.**
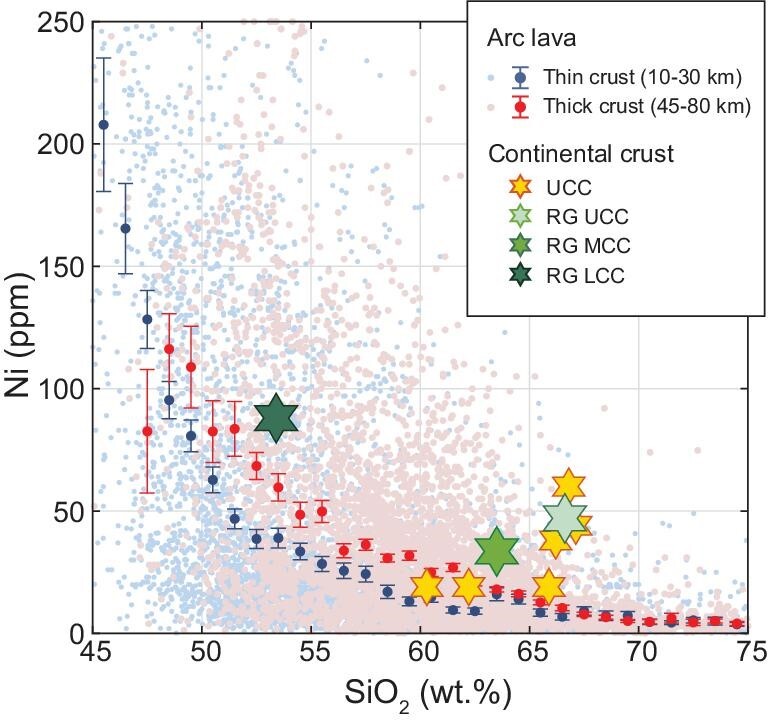
Ni-content evolution of arc lavas. Various estimates of Ni contents of the continental crust are plotted for comparison. These crustal composition estimates are from the same sources as those plotted in Fig. 1. Bin width 1 wt% SiO_2_.

The Ni content of the continental crust has also been used to argue for slab-melt/peridotite interactions in the generation of the continental crust (e.g. [5,54]). Rudnick and Gao [26] estimated somewhat higher Ni contents in the continental crust, particularly the upper continental crust, than arc lavas of similar SiO_2_ contents. However, unlike Mg#, the estimated Ni contents of the upper continental crust are highly variable and some are lower than in andesitic arc lavas (Fig. 5). This uncertainty compromises Ni as a useful tracer of slab-melt/peridotite interactions in continental crust formation based on current observations.

### The role of magma mixing/recharge

Mixing between basaltic and granitic magmas can also generate andesites with high Mg#. This is shown by the apparent geochemical mixing trends and zoned phenocrysts found in high-Mg# andesites from several localities [55–57]. Nevertheless, the higher P_2_O_5_ contents in andesites than in most arc basalts and granites suggest that andesites cannot form by mixing basaltic and granitic magmas in most cases [58]. In fact, concave/convex trends are commonly seen in major and trace element Harker diagrams such as Ni–SiO_2_ (Fig. 5), Al_2_O_3_–SiO_2_ and FeO_T_–MgO [59] for arc magma differentiation. These concave/convex differentiation trends are not consistent with magma mixing as the dominant process for arc magmas formation along the compositional spectrum because mixing would produce linear trends in element covariation diagrams.

More complex scenarios involving simultaneous magma recharge–fractionation have also been proposed to explain arc magma differentiation processes [60,61]. Extensive recharge tends to attenuate the depletions of compatible elements such as Mg and Fe^2+^ [60] and may fractionate Mg and Fe in evolved arc magmas if Mg and Fe have different compatibility. Magma recharge appears to be more efficient in thick-crust arcs [60,62], but Fe depletion becomes systematically enhanced with increasing crustal thickness [18,63], which is the opposite of what one would expect from magma recharge. This suggests that magma recharge alone cannot account for the behavior of compatible elements such as Mg and Fe in arc magma differentiation.

### Implications for continental crust formation

The Mg# of the continental crust only appears high, or anomalous, if one compares it with magmas from oceanic arcs. High-Mg# andesites form ubiquitously in continental arcs [64] and continental arc magmas have almost identical Mg# to the continental crust. This compositional difference between oceanic arc and continental arc magmas reflects the natural consequence of tholeiitic vs. calc-alkaline differentiation. In other words, the high Mg# of continental arc magmas and the continental crust results from Fe depletion instead of Mg addition.

Because calc-alkaline differentiation predominantly occurs in thick-crust arcs, synmagmatic crustal thickening appears to be key to continental crust formation [18,22,63]. Indeed, the upper continental crust has an average La/Yb of 15.4 [26], which is higher than that of average felsic magmas in oceanic arcs. The upper continental crust also has an elevated Dy/Yb ratio of ∼2 compared with felsic oceanic arc lavas (average Dy/Yb ∼1.6), which is a sign of garnet fractionation during continental crust formation. According to the empirical relationship between felsic rock La/Yb and crustal thickness from 16, the La/Yb of the upper continental crust indicates an average thickness of >50 km at the time of crust formation. While active arcs with crustal thickness of >50 km are less common today (mostly represented by the Northern and Central Andes), they are widely distributed in Earth's history. For example, Sierra Nevada, Kohistan, Gangdese, Coast Mountains and northern Mexican Cordillera were all once thickened to >50 km [16,65–67]. Today the preserved continental crust is ∼34 km on average [68]. The contrasting thickness between the preserved crust and the crust at the time of formation suggests that extensive thinning must have taken place after the formation of the continental crust, possibly due to lower crust recycling or erosion [67,69–72].

There is no doubt that subducted materials contribute to arc magmatisms [73]. In some places, there also appears to be irrefutable evidence for slab melting and generation of high-Mg# andesites in the mantle wedge [74]. However, our findings negate the need to invoke these processes as the dominant mechanism to explain the high Mg# of most continental arc magmas and the continental crust. The Mg# characteristics of the continental crust may be a natural consequence of intracrustal differentiation in thickened crust. On early Earth, hot subduction could have been more common [75,76], but even in the Archean, magmatic rocks show an average Mg#–SiO_2_ trend almost identical to that of mature continental arcs today [77]. Thereby, the chemical consequences of the igneous differentiation processes that formed the continental crust may have been similar over Earth's history.

## CONCLUSIONS

Magma Mg# differs between thick-crust continental arcs and thin-crust oceanic arcs. Andesites with Mg# of >0.5 are ubiquitous in continental arcs and continental arc magmas show an average Mg# similar to that of the continental crust. The pervasively high Mg# in continental arc magmas and the continental crust results from calc-alkaline intracrustal differentiation characterized by continuous Fe depletion. The comparable Mg# characteristics between the continental crust and continental arc magmas hint at synmagmatic crustal thickening as a critical process in the formation of the continental crust.

## Supplementary Material

nwac258_Supplemental_File
